# Test anxiety predictors inventory (tapi): development and initial validation of a predictor-oriented instrument for medical students

**DOI:** 10.1080/21642850.2026.2687929

**Published:** 2026-07-21

**Authors:** Dang Thanh Hong, Nguyen Thi Hong Duy, To Thi Kim Quyen

**Affiliations:** a Can Tho University of Medicine and Pharmacy, Can Tho city, Vietnam

**Keywords:** Test anxiety, scale development, medical students, academic stress, self-regulated learning

## Abstract

**Background:**

Test anxiety (TA) is common among medical students and may adversely affect mental well-being and academic performance. However, existing instruments such as the Test Anxiety Inventory (TAI) predominantly quantify the severity and manifestations of anxiety (outcomes) rather than identifying modifiable, intervention-relevant antecedents (predictors). This study aimed to develop and validate the Test Anxiety Predictors Inventory (TAPI) to support concurrent identification of at-risk students and to inform domain-specific, targeted interventions in resource-constrained medical training settings.

**Methods:**

A cross-sectional census survey was conducted at Can Tho University of Medicine and Pharmacy (Dec 2024–Mar 2025; 382/389 valid responses, 98.2%). A 30-item pool was piloted in 71 students, then refined through iterative EFA (3 rounds; ML extraction, Direct Oblimin) and CFA (ML and DWLS estimators). Internal consistency, convergent and discriminant validity, correlation with TAI, and concurrent classification performance (multivariable logistic regression; ROC-AUC) were assessed. Because census sampling yielded one dataset, EFA/CFA reflect internal testing pending external validation; ‘prediction’ denotes concurrent classification, not temporal forecasting.

**Results:**

TA prevalence (TAI ≥ 48) was 67.0% (95% CI 62.15–71.54). The final 14-item TAPI loaded on three factors—EAS (7), AMF (4), SRP (3)—explaining 77.99% of variance (KMO = 0.935). The correlated three-factor CFA showed good fit (CFI = 0.957; RMSEA = 0.083). The second-order DWLS model showed excellent fit but produced a Heywood warning with negative latent variance for EAS; the correlated three-factor model is therefore the more defensible representation. Reliability was high (α/ω = 0.89–0.95; CR = 0.92–0.96; AVE = 0.74–0.82; HTMT < 0.85). TAPI–TAI correlation was r = 0.576; classification model AUC = 0.804, sensitivity = 0.938, specificity = 0.452.

**Conclusions:**

TAPI shows promising internal structure and concurrent classification performance. High sensitivity supports first-stage screening, while modest specificity indicates it should complement, not replace, formal diagnostic procedures.

## 1. Introduction

Test anxiety (TA) impairs academic performance, mental health, and professional competence among medical students worldwide (Braier-Lorimer & Warren-Miell, 2022). Chronic academic stress in medical training is a key antecedent. Meta-analytic evidence shows anxiety affects 33.8% of medical students, exceeding rates in the general population and other disciplines (Quek et al., 2019). TA is a syndrome involving maladaptive physiological, behavioural, and cognitive responses before, during, and after assessment (Khaira et al., 2024). Cognitively, anxiety disrupts working memory and executive control, impairing concentration and knowledge retrieval. TA is associated with burnout, sleep disturbance, depressive symptoms, low self-esteem, and suicidal ideation (Eysenck et al., 2023). Unrecognised TA may reduce empathy, professionalism, and increase errors (Shafiee et al., 2024).

In Vietnam, this burden may be rising under integrated curricula, as students must manage clinical knowledge and achievement-related sociocultural and family pressures (Duong et al., 2025). Vietnamese studies emphasise strengthening preclinical skills to reduce academic stress in integrated programmes (Hong et al., 2025). Spielberger's Test Anxiety Inventory (TAI) remains the dominant measure of test anxiety (TA); however, it captures severity and overt manifestations—subjective distress, physiological arousal, worry, and failure-related cognitions—rather than the antecedents or contextual sources that drive anxiety in contemporary medical education (Idoiaga-Mondragon et al., 2025; Putwain et al., 2021). Developed for traditional examinations over four decades ago, it also inadequately reflects pressures from contemporary multidimensional assessments such as the OSCE and direct clinical observation (von der Embse et al., 2021), and instruments originating in Western contexts may not adequately capture culturally sensitive, multidimensional predictors of TA relevant to East Asian norms and integrated medical curricula (Nomura et al., 2023). Because current frameworks view test anxiety as arising from interactions among workload, learning environment, social expectations, and self-regulation, symptom-focused reliance on the TAI constrains predictor identification and limits the design of targeted, mechanism-informed interventions.

Addressing this gap requires an instrument that not only quantifies TA severity but also identifies modifiable psychosocial and behavioural antecedents to inform educational intervention, assessment redesign, clinical learning environment improvement, and psychological support for adaptive coping. In health professions education, early identification of at-risk students and domain-specific support may reduce academic underperformance and training burden (McCormick & Lamberson, 2024), consistent with health psychology models emphasising modifiable risks, self-regulation, and behavioural medicine for well-being (Pachón-Basallo et al., 2021). The Test Anxiety Predictors Inventory (TAPI) was therefore developed to shift medical education psychology from symptom description toward antecedent-domain identification (Zheng et al., 2023), integrating sociocultural pressures, academic environment, and learner characteristics—family expectations, integrated curriculum demands, motivation, and perseverance—into a multidimensional, context-sensitive framework that supports mechanism-focused research, screening, risk stratification, and locally tailored interventions (Ito et al., 2025). Importantly, TAPI domains are defined as risk-related appraisals rather than diagnostic markers: examination stress may resemble anxiety phenomenology, yet functions here as an antecedent vulnerability condition, not a defining criterion, positioning TAPI as an antecedent-oriented vulnerability profile for medical training rather than another measure of anxiety quantification.

This study integrates two frameworks explaining anxiety, performance, and adaptation to academic pressure. Attentional Control Theory posits that anxiety weakens goal-directed attention, heightens stimulus-driven processing, and diverts working-memory resources to intrusive thoughts rather than learning tasks (Yılmazer et al., 2024). Academic resilience, defined as sustaining motivation and achievement under adversity (Abdelrahman et al., 2025), functions as a protective mechanism in medical training by promoting adaptive cognitive–behavioural strategies and reducing stress-related harm to mental health and academic performance outcomes (Yusefi et al., 2025).

**Figure 1.**
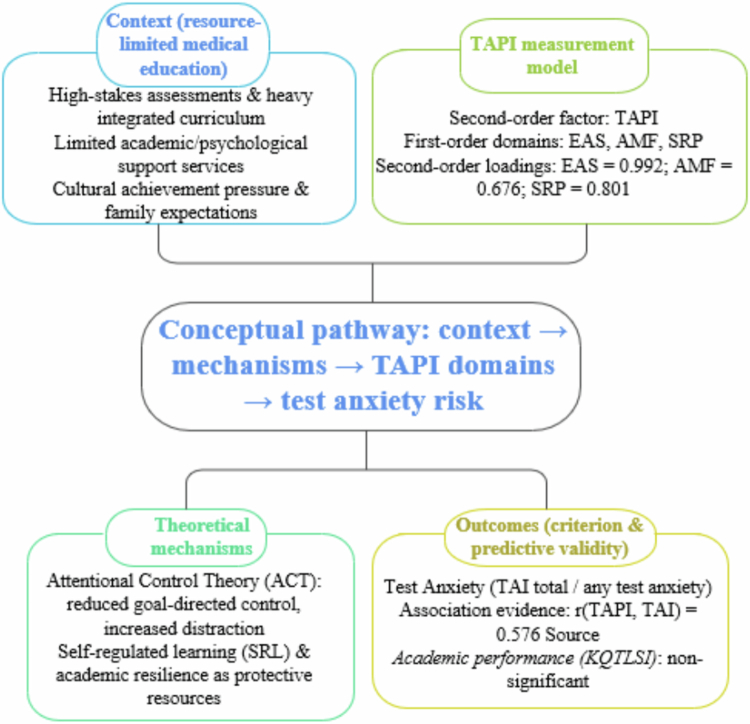
Conceptual framework for the development and validation of the Test Anxiety Predictors Inventory (TAPI).

EAS reflects appraised examination demands and academic-stress antecedents (not somatic/cognitive anxiety symptoms), whereas AMF and SRP index adaptive resources and self-regulatory capacity during preparation. Together, these domains form the higher-order TAPI construct statistically associated with concurrent test-anxiety status, as illustrated in Figure 1.

This study aimed to identify the TAPI factor structure through EFA, examine its multidimensional measurement model through internal CFA-based model testing, evaluate key psychometric properties, and assess concurrent classification performance for TAI-defined test anxiety using multivariable logistic regression.

## 2. Methods

### 2.1. Study design

This cross-sectional study followed the STROBE Statement and COSMIN recommendations, with transparent reporting of methodological decisions and content-validation limitations (Vandenbroucke et al., 2014). Conducted at Can Tho University of Medicine and Pharmacy from December 2024 to March 2025, it used census sampling of eligible medical students. Of 389 eligible students, 382 provided valid responses (98.2%). Before data collection, the questionnaire was piloted in 71 participants to assess clarity and reliability (Artino et al., 2014). Internal consistency was evaluated using Cronbach’s alpha (Tavakol & Dennick, 2011).

### 2.2. Participants and setting

Medical students enroled during the study period were recruited using census sampling. Inclusion criteria were current enrolment in the Doctor of Medicine programme, survey accessibility, informed consent, and questionnaire completion. Exclusion criteria were absence, inaccessibility, refusal, withdrawal, or invalid responses, defined as >10% missing data, straight-lining, or missing administrative information. Of 389 eligible students, 382 valid responses (98.2%) were analysed. Subgroup distributions by year of study, and other participant characteristics are reported in the descriptive tables to support interpretation of subgroup estimates. Duplicate and poor-quality records were screened before analysis; incomplete records were handled by listwise deletion. Listwise deletion excluded missing or invalid records; retained data were assumed adequate for stable estimation, but bias remains if missingness was nonrandom.

### 2.3. Instrument development

#### 2.3.1. Conceptualisation and item generation

TAPI identifies modifiable antecedents of test anxiety rather than severity or symptoms. A supplementary item-tracking table documents all 30 items, retention or deletion stages, and the conceptual and statistical grounds for removal. Given inconsistent evidence for anxiety-focused interventions, actionable predictors may better guide tiered academic support in resource-constrained settings for medical students (Williamson et al., 2024).

The TAPI framework was informed by international literature and Attentional Control Theory, integrating links among anxiety, attentional control, performance, motivation, and self-regulation (Eysenck et al., 2007). Three domains were specified: examination-related academic stress (EAS), adaptive motivation/focus (AMF), and self-regulated preparation (SRP), yielding a 30-item self-report pool (Appendix A) with clear, action-oriented wording. Provisional items were a priori assigned to three hypothesised domains, with overlap retained to refine breadth and redundancy. Item removal followed psychometric and conceptual criteria. Without cognitive interviews, structured pilot feedback from students was used to address wording and comprehension. Consistent with medical education scale-development principles, items were refined through learner feedback and EFA/CFA evidence to support risk stratification and domain-targeted intervention, with content validity explicitly prioritised under COSMIN (Artino et al., 2014).

#### 2.3.2. Expert review and content validity (CVI/CVR): transparent reporting in line with COSMIN

Under COSMIN, content validity is the foundational measurement property, requiring systematic evaluation of relevance, comprehensiveness, and comprehensibility with input from experts and target users in the intended context (Terwee et al., 2018). However, TAPI development lacked formal quantitative expert-panel validation, such as CVI/CVR or Delphi procedures; consequently, indices including I-CVI, S-CVI, and CVR were unavailable.

Thus, evidence for TAPI content appropriateness remains preliminary, supported by literature-based concept specification across EAS, AMF, and SRP, pilot assessment of clarity/comprehensibility, and item-refinement audit trails from EFA/CFA. This approach accords with medical education questionnaire-development guidance emphasising learner feedback and pilot testing to reduce measurement error (Artino et al., 2014). Future multicenter studies should undertake formal COSMIN-based content validation with both experts and medical students. Thus, this study represents TAPI’s initial development phase, emphasising internal structure and concurrent validity, while formal content validation remains pending.

#### 2.3.3. Cognitive pre-testing/pilot testing

Before the main survey, the draft questionnaire was piloted in 71 students with comparable characteristics to assess wording clarity, comprehensibility, interpretability, and internal consistency. This follows medical education guidance emphasising pretesting to identify wording, response-distribution, and item-quality problems before data collection (Artino et al., 2014).

Pilot testing showed strong reliability for the three initial clusters (EAS, AMF, and SRP), with Cronbach’s alpha values of 0.958, 0.917, and 0.871, supporting progression to the main survey. Alpha was appropriate for preliminary internal consistency assessment, although interpretation should consider scale structure and subsequent validity evidence (Tavakol & Dennick, 2011). Pilot feedback improved wording clarity and reduced interpretive inconsistency while preserving item meaning, consistent with pretesting recommendations in medical education (Willis & Artino, 2013).

#### 2.3.4. Iterative psychometric refinement and internal structural evidence

Iterative refinement across three EFA and four CFA rounds reduced 30 items to a 14-item, three-factor TAPI. Loadings exceeded 0.70, inter-factor correlations supported discriminant validity, and the scale balanced robustness with practical feasibility in medical education (Henseler et al., 2015). TAPI addresses the need for reliable tools capturing modifiable psychological and educational risk factors in medical education, enabling early screening, targeted support, and context-sensitive intervention design.

### 2.4. Data collection

Data were collected using a structured questionnaire under standardised procedures. Response submissions were monitored in real time to achieve the target sample and reduce missingness. After collection, the dataset was exported, cleaned, and screened; duplicate responses and poor-quality or non-serious submissions were excluded before analysis.

### 2.5. Data analysis

Analyses aligned with study objectives. Descriptive statistics summarised TAPI total and subscale scores; TAI-defined anxiety prevalence was reported with Wilson 95% confidence intervals. Factorability was evaluated using the Kaiser–Meyer–Olkin index and Bartlett’s test. EFA used maximum likelihood extraction with direct oblimin rotation; factor and item retention were based on eigenvalues, scree plot, interpretability, primary loadings, and cross-loadings. CFA compared one-factor, correlated three-factor, and second-order models using CFI, TLI, RMSEA, and SRMR. Maximum likelihood estimated first-order models, whereas DWLS was used for the second-order model. Because census sampling yielded a single dataset (*N* = 382), CFA served as internal model testing, not internal structural evaluation, and structural conclusions remain provisional pending replication in independent samples. Reliability and validity were assessed using Cronbach’s alpha, McDonald’s omega, CR, AVE, and HTMT. Known-groups validity used ANOVA with Tukey’s HSD and η². TAI severity categories served as a pragmatic external grouping criterion, providing supportive criterion-related evidence commonly used in test-anxiety research, though not fully independent validation. Classification performance used uni- and multivariable logistic regression, reporting odds ratios and 95% CIs. Model performance included AIC, BIC, pseudo-R², AUC, accuracy, sensitivity, specificity, confusion matrix, and multicollinearity diagnostics. Two-sided *p* < 0.05 indicated significance.

### 2.6. Ethics statement

This study was conducted as part of a university-level Science and Technology Project at Can Tho University of Medicine and Pharmacy and was approved by the Institutional Ethics Committee of Can Tho University of Medicine and Pharmacy under Decision No. 24.074.GV.

## 3. Results

### 3.1. Descriptive statistics

**Table 1.** Descriptive statistics of the Test Anxiety Predictors Inventory (TAPI) and its subscales.

Scale	K (number of items)	Theoretical range	Actual range	Mean (SD)	Skewness	Kurtosis
TAPI Total	14	14−70	14−70	47.44 (11.23)	−.514	.836
EAS (Examination Academic Stress)	7	7−35	7−35	24.45 (6.34)	−.44374	.191
AMF (Adaptive Motivation and Focus)	4	4−20	4−20	13.52 (3.47)	−.406	.320
SRP (Self-Regulated Preparation)	3	3−15	3−15	9.46 (3.24)	−.177	−.570

Table 1 presents the descriptive statistics for the TAPI (14 items) and its three subscales: EAS (7 items), AMF (4 items), and SRP (3 items). The mean total TAPI score was 47.44 ± 11.23 on a scale ranging from 14 to 70, with the observed range fully matching the theoretical range, indicating that the instrument captured the full score spectrum in the study sample. The mean subscale scores were 24.45 ± 6.34 for EAS (range 7–35), 13.52 ± 3.47 for AMF (range 4–20), and 9.46 ± 3.24 for SRP (range 3–15). For all subscales, the observed ranges were identical to the theoretical ranges, suggesting no apparent floor or ceiling effects at the descriptive level. Distributional indices showed slight negative skewness across all scales (TAPI = −0.514; EAS = −0.444; AMF = −0.406; SRP = −0.177), while kurtosis values remained within acceptable limits (TAPI = 0.836; EAS = 0.191; AMF = 0.320; SRP = −0.570).

**Table 2.** Prevalence of test anxiety (TAI) categories.

TAI category	TAI score range	n	Percentage	95% CI for percentage (Wilson)
No test anxiety	20−47	126	33.0	28.46–37.85
Low test anxiety	48−59	113	29.6	25.23–34.34
Moderate test anxiety	60−68	81	21.2	17.40–25.58
High test anxiety	69−80	62	16.2	12.87–20.26
**Any test anxiety (low–high)**	48−80	256	67.0	62.15–71.54

Table 2 shows that 67.0% of students had test anxiety (combining low to high levels; TAI 48–80), with a 95% Wilson confidence interval of 62.15–71.54, indicating a substantial burden of test anxiety in the study sample. The distribution by severity showed that 33.0% had no anxiety (20–47), whereas 29.6% had low anxiety (48–59), 21.2% had moderate anxiety (60–68), and 16.2% had high anxiety (69–80). The corresponding 95% Wilson confidence intervals provide an estimate of the precision of these proportions and support the use of a binary outcome variable, ‘any test anxiety,’ in predictive analyses.

### 3.2. Exploratory factor analysis

**Table 3.** Exploratory factor analysis pattern matrix for the Test Anxiety Predictors Inventory (TAPI): factor loadings, communalities, and uniqueness (*N* = 382).

Item code	Abbreviated item content	F1 (EAS)	F2 (AMF)	F3 (SRP)	h²	u²
**EAS1**	Worry about failing exam	.923			.760	.240
**EAS2**	Physical symptoms before exam	.834			.718	.282
**EAS3**	Intrusive worry during exam	.884			.809	.191
**EAS4**	Family/peer performance pressure	.705			.643	.357
**EAS5**	Fear of consequences of poor grades	.726			.718	.282
**EAS6**	Pre-exam sleep disturbance	.730			.683	.317
**EAS7**	Cognitive overload under exam conditions	.759			.706	.294
**AMF1**	Goal-directed study motivation		.810		.725	.275
**AMF2**	Sustained concentration during study		.958		.830	.170
**AMF3**	Self-confidence in academic ability		.740		.692	.308
**AMF4**	Positive reappraisal of exam challenge		.617		.527	.473
**SRP1**	Structured revision planning			−.845	.696	.304
**SRP2**	Self-monitoring of exam readiness			−.769	.777	.223
**SRP3**	Time management for exam preparation			−.804	.837	.163

Table 3 shows excellent factorability, with a KMO of 0.935 and a significant Bartlett’s test of sphericity (χ²(91) = 4476.29, *p* < 0.001; *N* = 382, listwise deletion). EFA extracted three factors matching the hypothesised TAPI domains: EAS, AMF, and SRP. All 14 items satisfied retention criteria, with primary loadings |λ| ≥ 0.60, cross-loading differences ≥0.20, and no salient cross-loadings, supporting clear subscale unidimensionality. Loading magnitudes were strong: 0.705–0.923 for EAS, 0.617–0.958 for AMF, and 0.769–0.845 for SRP in absolute values. Negative SRP loadings likely reflect arbitrary factor orientation under oblique rotation, not reverse scoring or conceptual inconsistency, given strong loadings and coherence within self-regulated preparation. Communalities were acceptable (0.527–0.837), with uniqueness values of 0.163–0.473, indicating substantial explained variance. Although AMF4 showed the lowest communality, it remained acceptable given the scale’s brevity and theoretical coherence. These results indicate a stable and interpretable three-factor solution overall.

### 3.3. Factor structure and CFA

**Table 4.** Confirmatory factor analysis model fit indices for TAPI scale validation (medical subsample, *N* = 382).

Model	Specification	Estimator	χ²	df	p	CFI	TLI	RMSEA (90% CI)	SRMR
Model 1	1-factor (TAPI; 14 items)	ML	817	35	<.001	0.717	0.636	0.242 (0.228–0.256)	0.114
Model 2	3 correlated factors (EAS: 7; AMF: 4; SRP: 3)	ML	267	74	<.001	0.957	0.947	0.083 (0.072–0.093)	0.041
Model 3	2nd-order (TAPI → EAS, AMF, SRP)	DWLS	299	74	<.001	0.998	0.998	0.062 (0.054–0.069)	0.033

Table 4 indicates poor fit for the one-factor model (CFI = 0.717; TLI = 0.636; RMSEA = 0.242; SRMR = 0.114), rejecting TAPI unidimensionality. The correlated three-factor first-order model showed good fit (CFI = 0.957; TLI = 0.947; RMSEA = 0.083; SRMR = 0.041), supporting the hypothesised multidimensional structure. The second-order model, estimated with DWLS for ordinal indicators, demonstrated excellent fit (CFI/TLI = 0.998; RMSEA = 0.062; SRMR = 0.033), supporting TAPI as a higher-order construct integrating EAS, AMF, and SRP.

Because TAPI items were ordinal Likert indicators, the second-order model was estimated using DWLS. Estimation produced a technical warning of negative latent variance, suggestive of a Heywood case, likely driven by the high second-order loading of EAS (≈0.99). Overall, findings support a coherent three-domain structure, but not strong reliance on the higher-order solution. Although theoretically appealing, the correlated three-factor model remains statistically preferable, pending replication in independent samples using robust ordinal estimators such as WLSMV at this stage.

### 3.4. Reliability

**Table 5.** Reliability and convergent validity of the TAPI measurement model.

Construct	Items	Cronbach's α	McDonald's ω	CR	AVE
AMF	4	0.891	0.892	0.921	0.744
EAS	7	0.945	0.945	0.960	0.775
SRP	3	0.904	0.907	0.929	0.815

Table 5 shows high internal consistency and strong convergent validity across all TAPI subscales. Cronbach’s *α* and McDonald’s *ω* were consistently high (AMF: *α* = 0.891, *ω* = 0.892; EAS: *α* = 0.945, *ω* = 0.945; SRP: *α* = 0.904, *ω* = 0.907), indicating substantial item homogeneity. Composite reliability also exceeded acceptable thresholds (AMF = 0.921; EAS = 0.960; SRP = 0.929), supporting stability within the factor structure. AVE values were high (0.744–0.815), showing that latent constructs explained substantial indicator variance and supporting convergent validity for EAS, AMF, and SRP.

**Table 6.** Discriminant Validity Assessment using Heterotrait-Monotrait Ratio (HTMT).

Construct Pair	HTMT
AMF–EAS	0.659
AMF–SRP	0.49
EAS–SRP	0.754

Table 6 shows HTMT ratios in the low-to-moderate range (AMF–EAS = 0.659; AMF–SRP = 0.490; EAS–SRP = 0.754), all below common thresholds, supporting discriminant validity and the multidimensional TAPI structure. The EAS–SRP pair showed the greatest overlap, but remained acceptable. Measurement invariance across academic performance groups was supported at configural, metric, scalar, and strict levels, with consistently excellent fit (CFI = 0.996), indicating a stable factor structure across subgroups. Detailed results are provided in Appendix B.

### 3.5. Validity evidence

Table 7 demonstrates strong known-groups validity: total TAPI scores increased progressively across test-anxiety categories, with significant differences and a large effect size (F(3,378) = 50.56, *p* < 0.001, η² = 0.286). Mean (SD) scores were 40.28 (10.54) for no anxiety, 46.75 (6.75) for low anxiety, 50.74 (9.87) for moderate anxiety, and 57.61 (11.10) for high anxiety. Post hoc tests confirmed an ordered gradient (1 < 2 < 3 < 4), consistent with the theoretical model.

**Table 7.** Known-groups validity: TAPI scores by test anxiety severity levels (*N* = 382).

Scale	TAI category	n	Mean (SD)	F (df1, df2)	p	η²	Post-hoc comparisons^a^
**TAPI_Total**	No anxiety (1)	126	40.28 (10.54)	50.56 (3, 378)	<.001	0.286	1 < 2 < 3 < 4
	Low anxiety (2)	113	47.48 (6.75)				
	Moderate anxiety (3)	81	50.74 (9.87)				
	High anxiety (4)	62	57.61 (11.10)				
**EAS**	No anxiety (1)	126	19.99 (5.55)	66.39 (3, 378)	<.001	0.345	1 < 2 < 3 < 4
	Low anxiety (2)	113	24.72 (4.14)				
	Moderate anxiety (3)	81	26.11 (5.36)				
	High anxiety (4)	62	30.87 (5.68)				
**AMF**	No anxiety (1)	126	12.55 (3.72)	7.85 (3, 378)	<.001	0.059	1 < 2, 3, 4^b^
	Low anxiety (2)	113	13.42 (2.48)				
	Moderate anxiety (3)	81	14.12 (3.25)				
	High anxiety (4)	62	14.90 (4.13)				
**SRP**	No anxiety (1)	126	7.74 (2.89)	32.36 (3, 378)	<.001	0.204	1 < 2 < 3 < 4
	Low anxiety (2)	113	9.34 (2.56)				
	Moderate anxiety (3)	81	10.51 (2.87)				
	High anxiety (4)	62	11.84 (3.46)				

^a^ Post-hoc comparisons using Tukey HSD, all pairwise *p* < .001 unless noted.

^b^ No anxiety group significantly lower than all other groups; groups 2–4 did not differ significantly from each other (*p* > .05).

EAS showed the strongest discriminative capacity across anxiety groups (F(3,378) = 66.39, *p* < 0.001, η² = 0.345), with mean scores increasing from 19.99 (5.55) to 24.72 (4.14), 26.11 (5.36), and 30.87 (5.68), and post hoc tests confirming a gradient (1 < 2 < 3 < 4). SRP also discriminated well (F(3,378) = 32.36, *p* < 0.001, η² = 0.204), increasing from 7.74 (2.89) to 11.84 (3.46) across groups. AMF showed smaller but significant differences (F(3,378) = 7.85, *p* < 0.001, η² = 0.059), suggesting a threshold rather than linear pattern.

### 3.6. Concurrent classification performance

**Table 8.** Univariable and multivariable logistic regression predicting any test anxiety among medical students (*N* = 382).

Predictor	Crude OR (95% CI)	Crude *p*	Adjusted OR (95% CI)	Adjusted *p*
**TAPI_Total** (per 1-point increase)	1.11 (1.08–1.14)	<.001	1.12 (1.09–1.15)	<.001
**Age** (years)	0.97 (0.71–1.32)	.838	0.94 (0.63–1.41)	.764
**Academic performance** (continuous)	0.76 (0.54–1.09)	.133	0.80 (0.52–1.22)	.302
**Sex** (female vs male)	1.27 (0.83–1.94)	.279	1.09 (0.66–1.78)	.746
**Exam timing** (afternoon vs morning)	0.66 (0.42–1.02)	.063	0.70 (0.42–1.17)	.172
**Ethnicity**				
- Tày vs Kinh	1.68 (0.65–4.32)	.283	1.77 (0.59–5.36)	.310
- Khmer vs Kinh	6.36 (0.82–49.48)	.077	15.22 (1.24–186.37)	.033
- Other vs Kinh	1.06 (0.35–3.18)	.916	1.04 (0.27–3.99)	.956
**Residence**				
- On-campus vs with family	0.89 (0.46–1.71)	.724	1.45 (0.66–3.21)	.358
- Rental housing vs with family	1.13 (0.52–2.46)	.763	1.65 (0.29–9.34)	.570
**Training programme** (integrated vs traditional)	0.90 (0.44–1.83)	.770	0.98 (0.42–2.30)	.960
**Household size**				
- 4 persons vs ≤ 3	1.06 (0.66–1.70)	.812	1.09 (0.61–1.93)	.781
- 5 persons vs ≤ 3	0.82 (0.29–2.35)	.714	0.60 (0.17–2.17)	.433
- ≥6 persons vs ≤ 3	0.90 (0.42–1.93)	.794	0.55 (0.10–2.91)	.478

Elevated TAPI scores were associated with increased odds of concurrent TAI-defined test anxiety in both univariable and multivariable analyses, supporting a classification-focused rather than temporally predictive interpretation. The observed AUC and high sensitivity indicate potential practical value for first-stage triage, whereas modest specificity suggests that TAPI should prompt further evaluation rather than support stand-alone decisions about individual students. The ethnicity-related association should be interpreted as exploratory only, because the confidence interval was very wide and the relevant subgroup estimates were likely unstable (Table 8).

The overall performance of the adjusted logistic regression model is presented in Table 9.

**Table 9.** Model performance, discrimination, and classification metrics of the adjusted logistic regression model (*N* = 382).

A. Model Fit Indices	
Metric	Value
Deviance	387.0
Akaike Information Criterion (AIC)	417.0
Bayesian Information Criterion (BIC)	476.0
McFadden's pseudo-R²	0.202
Cox & Snell R²	0.226
Nagelkerke R²	0.314
Tjur's R²	0.248
Overall model χ² (df)	97.8 (14)
Overall model *p*-value	<.001
The adjusted logistic model showed significant fit (χ²(14) = 97.8, *p* < 0.001), outperforming the null model. Explained variation was moderate (McFadden’s pseudo-R² = 0.202; Cox and Snell R² = 0.226; Nagelkerke R² = 0.314; Tjur’s R² = 0.248), indicating acceptable explanatory and discriminative capacity. Information criteria (AIC = 417.0; BIC = 476.0; deviance = 387.0) further supported model comparison.	

B. Discrimination and Classification Performance	
Metric	Value
Area Under the ROC Curve (AUC)	0.804
Overall accuracy	0.777
Sensitivity (true positive rate)	0.938
Specificity (true negative rate)	0.452
Positive Predictive Value (PPV)	0.777
Negative Predictive Value (NPV)	0.780
Classification cut-off (probability threshold)	0.50
The model showed good discrimination (AUC = 0.804), indicating performance beyond chance in distinguishing students with and without test anxiety. At the 0.50 threshold, accuracy was 0.777 and sensitivity 0.938, whereas specificity was lower (0.452). Thus, the model is better suited for detection and early screening than rule out classification. PPV (0.777) and NPV (0.780) indicated moderate classification reliability in this sample with high anxiety prevalence.	

C. Confusion Matrix (Cut-off = 0.50)			
	Predicted: No anxiety	Predicted: Any anxiety	% Correctly classified
**Observed: No anxiety (*n* = 126)**	57	69	45.2%
**Observed: Any anxiety (*n* = 256)**	16	240	93.8%
**Overall (*N* = 382)**	**73**	**309**	**77.7%**
The classification model achieved 77.7% accuracy. Of 256 students with test anxiety, 240 (93.8%) were correctly identified, with 16 false negatives, indicating high sensitivity. By contrast, specificity was low: only 57 of 126 students without anxiety (45.2%) were correctly classified, whereas 69 were false positives. This pattern is consistent with screening-oriented use prioritising case detection. For individual-level classification, threshold adjustment or optimisation by Youden’s J or sensitivity–specificity balance should therefore be considered carefully.			

D. Multicollinearity Diagnostics		
Predictor	VIF	Tolerance
TAPI_Total	1.03	0.970
Age	1.01	0.986
Academic performance	1.06	0.940
Sex	1.01	0.992
Exam timing	1.02	0.979
Ethnicity	1.06	0.942
Residence	1.43	0.701
Household size	1.31	0.763
Training programme	1.01	0.988
Multicollinearity diagnostics indicated no meaningful concern: all variance inflation factors were low (1.01–1.43) and tolerance values were high (0.701–0.992), suggesting minimal informational overlap among the predictors in the logistic model and relatively stable odds ratio estimates. The predictor with the highest VIF was residence (VIF = 1.43; tolerance = 0.701), which remained well below commonly used thresholds of concern (e.g. VIF > 5 or > 10). Accordingly, the model can be considered largely unaffected by multicollinearity.		

## 4. Discussion

### 4.1. Summary of key findings

This study developed and validated the Test Anxiety Predictors Inventory (TAPI), a brief instrument assessing modifiable psychosocial and behavioural predictors of test anxiety in medical students. TAPI addresses a gap in health psychology and behavioural medicine: existing measures mainly quantify anxiety severity rather than intervention-relevant antecedents. By capturing examination-related academic stress, adaptive motivation–focus, and self-regulated preparation, TAPI supports early risk screening and domain-specific support. Its main contribution is shifting assessment from anxiety outcomes to modifiable upstream domains that can inform targeted intervention and educational decision-making. This orientation aligns with medical education questionnaire-development guidance and COSMIN principles for conceptually grounded, valid instruments (Williamson et al., 2024). TAPI therefore measures antecedents rather than anxiety severity as an outcome (Eysenck et al., 2023).

Structurally, EFA and CFA showed that TAPI has a coherent, multidimensional organisation. EFA extracted three factors explaining 77.99% of variance, with Factor 1 dominant (eigenvalue = 8.107; 57.91%), and the remaining factors contributing 12.28% and 7.80%. Retention of the three-factor solution was supported by eigenvalues, scree plot inspection, and theoretical interpretability, favoring a multidimensional over unidimensional conceptualisation of test-anxiety predictors (Cosemans et al., 2022). Although examination-related academic stress was the central domain, adaptive motivation–focus and self-regulated preparation strengthened the instrument’s relevance for targeted intervention in medical education (Ballouk et al., 2022; Zhang et al., 2022).

TAPI showed known-groups validity and screening-oriented classification performance, but should not be interpreted as a diagnostic instrument. Strong item loadings (several ≥0.95 within EAS and AMF) and reliability coefficients (CR up to 0.960) support convergent validity but raise concerns of content redundancy and narrow construct coverage; future revisions should consider item-rewording or trimming to broaden domain breadth. Likewise, TAI-group comparisons provide supportive criterion-related evidence, not independent validation, because grouping relied on an established anxiety instrument. Scores increased across test-anxiety levels, supporting validity within COSMIN frameworks (Mokkink et al., 2024). The classification model, interpreted as concurrent statistical association and high sensitivity, supporting early detection, although specificity was limited. TAPI is therefore best used for initial screening and support stratification, followed by further contextual assessment before decisions, consistent with ROC/AUC principles and sensitivity–specificity trade-offs (Çorbacıoğlu & Aksel, 2023; Theobald et al., 2022).

### 4.2. Comparison with existing literature

The findings are consistent with the broader literature indicating that test anxiety is a prominent concern among medical students because of the demanding curriculum and the high-stakes nature of assessment, and that anxiety-reduction interventions do not necessarily translate into improved academic performance unless they target the underlying mechanisms, such as learning strategies, self-regulation, or the assessment environment. This helps explain the practical value of the TAPI’s predictor-oriented approach: rather than merely identifying who is anxious, the instrument is designed to clarify why anxiety increases and which domain should be targeted for intervention.

Linking EAS, AMF, and SRP to Attentional Control Theory (ACT) situates the findings within an explanatory framework. ACT proposes that anxiety weakens goal-directed attention and increases susceptibility to distraction, thereby impairing preparation and performance. It also offers an intervention logic: reducing examination pressure and uncertainty while strengthening concentration and self-regulation may disrupt the cycle of anxiety and ineffective preparation. Although much ACT evidence derives from experimental settings, its account of attentional imbalance plausibly explains how academic stress and reduced self-regulation heighten test anxiety.

Validity evidence based on external variables (Table S1) supports TAPI structural plausibility. The total TAPI score correlated moderately with total TAI (r = 0.576), whereas domain-specific associations varied (EAS: r = 0.615; SRP: r = 0.480; AMF: r = 0.291). This pattern suggests that examination-related academic stress is most directly linked to anxiety, while motivation–focus and self-regulation may represent actionable adaptive resources. Academic performance (KQTLSI) was not associated with either TAPI (r = 0.001) or TAI (r = −0.046). Rather than indicating no academic effect, this likely reflects timing, assessment format, or mediation by preparation behaviour, warranting longitudinal examination-specific studies.

### 4.3. Theoretical and practical implications

From a theoretical perspective, TAPI contributes by distinguishing between (i) anxiety as a state or symptomatic outcome, as captured by the TAI, and (ii) modifiable antecedent domains, represented by EAS, AMF, and SRP. This distinction is consistent with the view in medical education that measurement is most meaningful when it enables institutions to move beyond problem identification toward mechanism-informed intervention design. The CFA findings supporting both a multidimensional structure and a second-order model suggest that a general shared variance among predictor domains may underlie the TAPI, while preserving domain-specific meaning for targeted intervention.

From a practical perspective, the concurrent classification model's high sensitivity but limited specificity indicates that TAPI is well suited for early screening and support triage, where minimising missed at-risk cases is a priority. However, if the instrument is used to inform individual-level decisions, such as mandatory counselling referral, it should be clearly communicated that TAPI provides a risk assessment rather than a diagnosis, and that classification thresholds should be selected according to the intended purpose (e.g. screening versus diagnostic classification). At the 0.50 threshold, the model demonstrated high sensitivity but only modest specificity. To optimise educational utility in resource-constrained settings, we therefore propose a two-step screening approach. In the first step, TAPI functions as a broad-coverage triage-oriented screening instrument designed to identify students at potential risk. In the second step, students with positive screening results undergo confirmatory assessment using a standardised instrument, such as the TAI, or a brief academic or psychological consultation to refine support stratification and reduce the impact of false-positive classifications on the support system. This approach allows the strengths of TAPI, particularly its high sensitivity, to be leveraged while maintaining feasibility in real-world implementation.

At the programmatic level, the very weak associations with age and sex (*p* > 0.05) suggest that, in this sample, TAPI was not strongly driven by basic demographic characteristics. However, the small but statistically significant association between TAPI total and examination timing (r = −0.101; *p* = 0.049) indicates that the assessment context may influence how students report predictive factors. Accordingly, to improve data consistency and comparability across administrations, the measurement window should be standardised in future implementation, for example, by administering the instrument 1–2 weeks before examinations with a consistent response instruction format. TAPI may therefore be more appropriately conceptualised as a system-level tool for support stratification rather than as a fixed individual label.

Based on its domain structure, TAPI may also inform tiered intervention design. Although the TAPI domains were selected because they are theoretically modifiable and relevant to intervention planning, the present cross-sectional design does not establish causal influence or responsiveness to change. Students with high EAS scores may benefit most from programme- or assessment-level interventions, such as clearer assessment blueprints, more formative feedback, and reduced uncertainty around examinations. Those with low SRP scores may require support focused on self-regulation skills, including planning, time management, and self-monitoring. Students with low AMF scores may benefit from interventions aimed at strengthening self-efficacy and maintaining motivation and attentional focus. This domain-based implementation strategy may be particularly valuable in resource-limited settings because it allows support resources to be allocated according to the principle of matching the right intervention to the right group.

### 4.4. Implications for health psychology and behavioural medicine

The development of TAPI has several implications for health psychology research and practice in educational settings. First, by measuring theoretically modifiable risk-related domains rather than anxiety symptoms, TAPI aligns with the preventive and health-promotion orientation central to behavioural medicine. Students with high EAS scores may benefit from psychoeducation about stress management and cognitive reappraisal techniques; those with low SRP may require behavioural interventions targeting time management and self-monitoring skills; and students with low AMF may benefit from motivational interviewing or self-efficacy enhancement programmes.

Second, TAPI's high sensitivity (0.938) positions it as a viable first-stage triage-oriented screening instrument in tiered mental health support systems within medical schools. In resource-limited contexts where comprehensive psychological assessment may not be feasible for all students, TAPI can efficiently identify at-risk individuals for further evaluation using validated clinical instruments or brief counselling.

Third, the domain-based structure of TAPI provides a behavioural map for institutional quality improvement. Rather than treating test anxiety as a fixed individual trait, medical schools can use TAPI data to identify systemic stressors (e.g. assessment unpredictability, excessive workload) and implement programme-level interventions that reduce examination-related stress while strengthening students' psychological resources and self-regulatory capacity.

Although developed in Vietnamese medical education, TAPI assesses transferable risk mechanisms across high-pressure contexts, including stress appraisal, motivational regulation, and self-regulated preparation; cross-cultural multicenter validation remains essential before wider implementation.

### 4.5. Strengths and limitations

#### 4.5.1. Strengths

A key strength of this study is its logically sequenced psychometric evaluation, spanning factor structure, reliability, construct validity, known-groups validity, and predictive performance. The high response rate and inclusion of all eligible students reduced internal selection bias and strengthened contextual applicability.

#### 4.5.2. Limitations

Several limitations should be noted. First, the cross-sectional design does not permit causal inference; accordingly, ‘prediction’ refers to statistical classification rather than temporal prediction. Second, self-reported data may be influenced by social desirability and response context. Third, the second-order CFA showed a very high loading of TAPI on EAS (0.992), and DWLS estimation generated a negative latent variance warning, suggestive of a possible Heywood case. Although several indicator loadings were also very high, supporting convergent validity, these results require transparent reporting of model fit and clear justification for estimator choice for ordinal Likert-type data. Fourth, because the study was conducted at a single institution, generalisability to other medical schools or assessment contexts should be interpreted cautiously.

Another limitation is that EFA and CFA were conducted in the same sample, which may have optimised model fit to this study population; thus, the three-factor and second-order structures require confirmation in independent samples before broader implementation. Invariance across academic performance groups may provide supportive technical evidence, but should be interpreted cautiously because performance is shaped by multiple influences. Future studies should prioritise invariance testing across more stable grouping variables, such as sex, academic year, and assessment format. Finally, ethnic-group differences were associated with wide confidence intervals, likely reflecting small subgroup sizes, and should therefore be considered exploratory rather than definitive.

Key limitations include absent COSMIN-level content validation, same-sample EFA/CFA restricting structural confirmation, and a Heywood warning in second-order CFA, indicating that the higher-order model remains provisional and not yet definitive.

### 4.6. Future research directions

Future research should proceed in three main directions. First, longitudinal studies are needed to evaluate temporal classification performance by measuring TAPI before examinations and tracking test anxiety and academic outcomes in relation to specific assessments. Such designs would help clarify the mediating mechanisms linking examination-related stress, self-regulation, and test anxiety. Second, cross-validation across multiple institutions and academic years is required to assess generalisability and measurement invariance across key background subgroups. Third, intervention studies should be designed on the basis of domain-specific profiles (EAS, AMF, and SRP) to determine whether targeting the appropriate domain can reduce test anxiety and improve academic performance. This is particularly important given that anxiety-reduction interventions alone do not always translate into improved academic outcomes.

Longitudinal research should examine stability of the second-order structure and changes in EAS, AMF, and SRP before and after intervention, as these domains differ in their associations with TAI scores and may respond differently to support. Supplementary findings—correlations with TAI, invariance across academic-performance groups, and the high second-order loading of EAS—indicate the need to test temporal stability and parameter robustness across assessment contexts. Re-estimation with ordinal-data estimators such as WLSMV is warranted to assess sensitivity of the second-order model and clarify Heywood risk. If validated, TAPI could support quality assurance, well-being surveillance, and intervention planning.

## 5. Conclusions

TAPI offers encouraging preliminary evidence as a brief, predictor-oriented measure of risk-related domains linked to concurrent test-anxiety status among medical students. Based on current evidence, the correlated three-factor structure—rather than the higher-order solution—is endorsed as the operational measurement model of TAPI, given the Heywood warning and negative latent variance observed in the second-order CFA. Internal consistency was satisfactory and first-stage screening utility was supported by high sensitivity. Nevertheless, formal content validation, external replication, and longitudinal assessment remain necessary before TAPI can be recommended for wider implementation or interpreted as a stand-alone decision instrument.

## Supplementary Material

Appendix B.docx Appendix B.docx

Appendix A.docx Appendix A.docx

Appendix C.docx Appendix C.docx

SUPPLEMENTARY Table S1F.docx SUPPLEMENTARY Table S1F.docx

## Data Availability

The dataset used in this study is currently stored at Can Tho University of Medicine and Pharmacy and may be made available by the corresponding author upon reasonable request, subject to applicable ethical and data protection regulations.

## Author declaration statement

This manuscript has been read and approved by all named authors; DTH, NTHD, TTKQ. There are no other persons who satisfied the authorship criteria but are not listed. We thereby confirm that the order of the authors listed in this manuscript have been approved by all of us.
