# Supplementary material for: Test anxiety predictors inventory (tapi): development and initial validation of a predictor-oriented instrument for medical students
Source: Health Psychol Behav Med. 2026 Jul 21;14(1):2687929. doi: 10.1080/21642850.2026.2687929 (PMC13390163; doi:10.1080/21642850.2026.2687929)

Appendix B

| **KMO and Bartlett's Test** | | |
| --- | --- | --- |
| Kaiser-Meyer-Olkin Measure of Sampling Adequacy. | | .935 |
| Bartlett's Test of Sphericity | Approx. Chi-Square | 4476.286 |
|  | df | 91 |
|  | Sig. | .000 |

| **Total Variance Explained** | | | | | | | | | |
| --- | --- | --- | --- | --- | --- | --- | --- | --- | --- |
| Component | Initial Eigenvalues | | | Extraction Sums of Squared Loadings | | | Rotation Sums of Squared Loadings | | |
|  | Total | % of Variance | Cumulative % | Total | % of Variance | Cumulative % | Total | % of Variance | Cumulative % |
| 1 | 8.107 | 57.907 | 57.907 | 8.107 | 57.907 | 57.907 | 4.950 | 35.358 | 35.358 |
| 2 | 1.720 | 12.283 | 70.190 | 1.720 | 12.283 | 70.190 | 3.249 | 23.209 | 58.568 |
| 3 | 1.092 | 7.797 | 77.986 | 1.092 | 7.797 | 77.986 | 2.719 | 19.419 | 77.986 |
| 4 | .458 | 3.269 | 81.256 |  |  |  |  |  |  |
| 5 | .419 | 2.996 | 84.252 |  |  |  |  |  |  |
| 6 | .373 | 2.665 | 86.917 |  |  |  |  |  |  |
| 7 | .325 | 2.319 | 89.236 |  |  |  |  |  |  |
| 8 | .281 | 2.008 | 91.244 |  |  |  |  |  |  |
| 9 | .258 | 1.843 | 93.086 |  |  |  |  |  |  |
| 10 | .226 | 1.614 | 94.701 |  |  |  |  |  |  |
| 11 | .216 | 1.542 | 96.243 |  |  |  |  |  |  |
| 12 | .200 | 1.428 | 97.670 |  |  |  |  |  |  |
| 13 | .167 | 1.196 | 98.867 |  |  |  |  |  |  |
| 14 | .159 | 1.133 | 100.000 |  |  |  |  |  |  |
| Extraction Method: Principal Component Analysis. | | | | | | | | | |

| **Rotated Component Matrix^a^** | | | |
| --- | --- | --- | --- |
|  | Component | | |
|  | 1 | 2 | 3 |
| EAS1 | .840 |  |  |
| EAS3 | .820 |  |  |
| EAS2 | .795 |  |  |
| EAS7 | .766 |  |  |
| EAS5 | .758 |  |  |
| EAS4 | .745 |  |  |
| EAS6 | .736 |  |  |
| AMF2 |  | .877 |  |
| AMF1 |  | .824 |  |
| AMF3 |  | .815 |  |
| AMF4 |  | .747 |  |
| SRP1 |  |  | .879 |
| SRP3 |  |  | .807 |
| SRP2 |  |  | .806 |
| Extraction Method: Principal Component Analysis.  Rotation Method: Varimax with Kaiser Normalization. | | | |
| a. Rotation converged in 6 iterations. | | | |


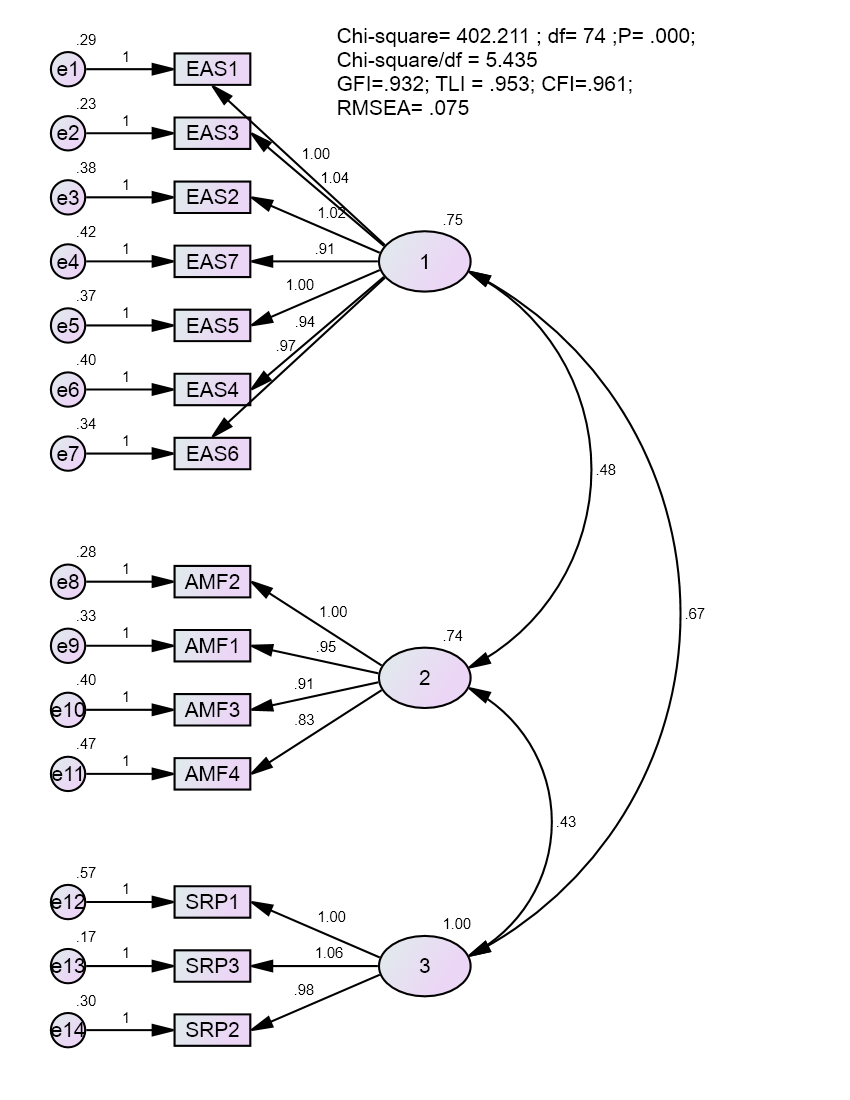

Supplement: Appendix B.docx [file RHPB_A_2687929_SM2472.docx]
