# Supplementary material for: Test anxiety predictors inventory (tapi): development and initial validation of a predictor-oriented instrument for medical students
Source: Health Psychol Behav Med. 2026 Jul 21;14(1):2687929. doi: 10.1080/21642850.2026.2687929 (PMC13390163; doi:10.1080/21642850.2026.2687929)
Supplement: Appendix A.docx [file RHPB_A_2687929_SM2468.docx]

**Appendix A:**

***Personal Factors (PERS)***

PERS 01. I often feel stressed or anxious without a clear reason.

PERS 02. I feel disappointed in myself when I do not get the results I expect.

PERS 03. When I feel anxious, I find it hard to calm myself down.

PERS 04. I usually make a detailed study plan before each exam.

PERS 05. I usually stick to my study plan.

PERS 06. I know how to prioritize important subjects or topics during exam preparation.

PERS 07. I find it difficult to balance study time and rest time.

PERS 08. I know how to prepare effectively for exams based on my past experience.

PERS 09. Poor results in previous exams make me anxious about future exams.

PERS 010. I feel more anxious when taking an exam in an unfamiliar format.

***Academic Factors (ACAD)***

ACAD011. I feel pressured when completing practical assignments in this course.

ACAD012. I worry that I may not be able to meet the requirements of this course.

ACAD013. The difficulty of this course makes me feel stressed during studying and exam preparation.

ACAD014. This course contains too much material to study and review.

ACAD015. The time available for this course is too short for me to master all the content.

ACAD016. The workload in this course makes me feel stressed.

***Environmental Factors (ENVR)***

ENVR017. My family’s expectations make me feel pressured when taking exams.

ENVR018. My family has very high expectations for my academic performance.

ENVR019. Comparing my academic performance with my peers makes me feel pressured.

ENVR020. I always want to score higher than my classmates.

ENVR021. I feel anxious when I think my peers may score higher than I do on an exam.

ENVR022. The way exams are organized (e.g., timing, procedures) makes me feel more stressed.

ENVR023. The exam room environment (e.g., lighting, air, temperature) makes it hard for me to concentrate.

***Health Factors (HLTH)***

HLTH024. Lack of sleep often leaves me tired and low in energy, making it hard to concentrate during exams.

HLTH025. When I do not feel physically well (e.g., due to lack of sleep or poor diet), I feel more anxious and stressed during exams.

***Emotional and Motivational Factors (EMOT)***

EMOT026. I am afraid that I will fail if I do not study well enough.

EMOT027. Fear of failure makes me anxious even when I have prepared well for an exam.

EMOT028. When studying this course, I feel that I am studying mainly to pass the exam rather than to understand the material.

EMOT029. Lack of motivation makes it hard for me to do well in this course.

EMOT030. When studying this course, I find it difficult to stay focused for a long time.
