# Supplementary material for: Test anxiety predictors inventory (tapi): development and initial validation of a predictor-oriented instrument for medical students
Source: Health Psychol Behav Med. 2026 Jul 21;14(1):2687929. doi: 10.1080/21642850.2026.2687929 (PMC13390163; doi:10.1080/21642850.2026.2687929)
Supplement: Appendix C.docx [file RHPB_A_2687929_SM2470.docx]

**Appendix C:**

**Vietnamese version:**

***Nhóm 1: Áp lực–căng thẳng thi cử (ALCT)***

1. Tôi cảm thấy áp lực khi thực hiện các bài tập thực hành trong môn học này

2. Độ khó của môn học này khiến tôi cảm thấy căng thẳng trong quá trình học tập và ôn thi

3. Tôi lo lắng rằng mình không đủ khả năng để đáp ứng các yêu cầu của môn học này

4. Tôi lo sợ rằng mình sẽ thi trượt nếu không học tập đủ tốt

5. Khi tôi cảm thấy không khỏe (do thiếu ngủ hoặc chế độ ăn uống kém), tôi dễ cảm thấy lo lắng và căng thẳng trong kỳ thi

6. Môn học này có quá nhiều nội dung cần học và ôn tập

7. Kết quả thi không tốt trước đây khiến tôi cảm thấy lo lắng trong các kỳ thi tiếp theo

***Nhóm 2: Động lực, tập trung trong bối cảnh áp lực (DLTT)***

8. Tôi thường lập kế hoạch học tập chi tiết trước mỗi kỳ thi

9. Tôi thường thực hiện đúng kế hoạch học tập mà mình đã đề ra

10. Tôi biết cách ưu tiên những môn học hoặc nội dung quan trọng hơn trong thời gian ôn thi

11. Tôi biết cách chuẩn bị hiệu quả cho các kỳ thi nhờ vào kinh nghiệm của mình

***Nhóm 3: Tự quản lý ôn thi và năng lực chuẩn bị (QLNL)***

12. Khi học môn học này, tôi cảm thấy mình chỉ học để đối phó với kỳ thi, không phải vì thực sự muốn hiểu bài

13. Thiếu động lực học tập khiến tôi cảm thấy khó đạt được kết quả tốt trong môn học này

14. Khi học môn học này, tôi gặp khó khăn trong việc tập trung và duy trì sự chú ý lâu dài

**English version:**

Group 1: Examination-related academic stress (EAS)

EAS01. I feel pressured when completing practical tasks in this course.

EAS02. The difficulty of this course makes me feel stressed during study and exam preparation.

EAS03. I worry that I may not be able to meet the requirements of this course.

EAS04. I am afraid that I will fail the exam if I do not study well enough.

EAS05. When I do not feel physically well (e.g., because of lack of sleep or poor diet), I feel more anxious during exams.

EAS06. This course contains too much material to study and review.

EAS07. Poor results in previous exams make me anxious about future exams.

Group 2: Adaptive motivation/focus (AMF)

AMF01. I usually make a detailed study plan before each exam.

AMF02. I usually stick to my study plan.

AMF03. I know how to prioritize important subjects or topics during exam preparation.

AMF04. I know how to prepare effectively for exams based on my past experience.

Group 3: Self-regulated preparation (SRP)

SRP01. When studying this course, I feel that I am studying mainly to pass the exam rather than to understand the material.

SRP02. Lack of motivation makes it hard for me to do well in this course.

SRP03. When studying this course, I find it difficult to stay focused for a long time.
