# Supplementary material for: Test anxiety predictors inventory (tapi): development and initial validation of a predictor-oriented instrument for medical students
Source: Health Psychol Behav Med. 2026 Jul 21;14(1):2687929. doi: 10.1080/21642850.2026.2687929 (PMC13390163; doi:10.1080/21642850.2026.2687929)
Supplement: SUPPLEMENTARY Table S1F.docx [file RHPB_A_2687929_SM2471.docx]

**SUPPLEMENTARY MATERIAL**

**Table S1.** Evidence of validity based on relations with other variables (N = 382)

| External variable | Type | Correlation with TAPI_Total | | | Correlation with TAI_total | | |
| --- | --- | --- | --- | --- | --- | --- | --- |
|  |  | r | 95% CI | p | r | 95% CI | p |
| Criterion validity |  |  |  |  |  |  |  |
| TAI_total (test anxiety) | Continuous | 0.576 | [0.504, 0.639] | <.001 | — | — | — |
| KQTLSI (academic performance) | Continuous | 0.001 | [−0.099, 0.101] | .987 | −0.046 | [−0.146, 0.054] | .365 |
| Convergent validity |  |  |  |  |  |  |  |
| EAS (Examination Academic Stress) | Continuous | 0.944 | [0.931, 0.954] | <.001 | 0.615 | [0.549, 0.674] | <.001 |
| AMF (Adaptive Motivation Focus) | Continuous | 0.768 | [0.724, 0.807] | <.001 | 0.291 | [0.196, 0.380] | <.001 |
| SRP (Self-Regulated Preparation) | Continuous | 0.798 | [0.759, 0.832] | <.001 | 0.480 | [0.399, 0.554] | <.001 |
| Discriminant validity |  |  |  |  |  |  |  |
| Age | Continuous | 0.034 | [−0.066, 0.134] | .502 | 0.021 | [−0.079, 0.121] | .681 |
| Sex | Binary | 0.052 | [−0.049, 0.151] | .312 | 0.055 | [−0.046, 0.154] | .285 |
| Exam timing (morning/afternoon) | Binary | −0.101 | [−0.199, −0.001] | .049 | −0.079 | [−0.178, 0.021] | .122 |
